# Editorial: Neurobiology of Peripartum Mental Illness

**DOI:** 10.3389/fgwh.2022.888088

**Published:** 2022-05-27

**Authors:** Jodi L. Pawluski, Gisèle Apter, Jayashri Kulkarni, Lauren M. Osborne

**Affiliations:** ^1^Univ Rennes, INSERM, EHESP, Irset (Institut de Recherche en Santé, Environnement et Travail), UMR_S 1085, Rennes, France; ^2^Faculté de Médecine et de Pharmacie, Université de Rouen, Groupe Hospitalier du Havre, Le Havre, France; ^3^Monash University, Melbourne, VIC, Australia; ^4^Department of Psychiatry & Behavioral Sciences, The Johns Hopkins University School of Medicine, Baltimore, MD, United States

**Keywords:** mental illness, postpartum, postpartum depression (PPD), pregnancy complications, peripartum, neuroscience, motherhood, neurobiology

Mental illness, both during pregnancy and beyond, has devastating consequence for parents, their children, and society at large. Perinatal depression affects 15–20% of mothers and birthing people and is often cited as the most common complication of childbirth, with suicide being a leading cause of death in the first year postpartum (1). Peripartum anxiety rates in some studies are even higher, with other peripartum mental illness (i.e., postpartum psychosis, peripartum obsessive-compulsive disorder) being less common but more serious (2). In fact the peripartum period has repeatedly been shown to be linked with an increased risked of developing a mental illness (3).

Psychological and social risk factors for peripartum mental illness are well established, but our understanding of the neurobiology associated with these illnesses is limited. Given that these are “mental” illnesses it is disappointing that we know so little about the brain mechanisms mediating them. Unpacking the biological mechanisms of peripartum mental illness would not only help us to understand and treat illnesses that wreak havoc for mothers and families, but would also help to pinpoint biological mechanisms that remain a mystery in so many mental illnesses.

The current Research Topic titled *Neurobiology of Peripartum Mental Illness* aimed to increase our understanding of the neural mechanisms mediating peripartum adaptations and peripartum mental illness by bringing together basic and clinical research through original manuscripts and reviews. This Research Topic covers a number of aspects of neuroscience in relation to peripartum mental illness and parenting, in general.

Recent research has led to our understanding that key structural brain changes accompany biological motherhood, and Martínez-García et al. cover a review of this recent research. They discuss proposed mechanisms behind these brain structural changes with pregnancy and birth and how these changes may have implications for peripartum mental health. The role of the immune system in peripartum mental illness has recently become quite an interesting topic of investigation, and the review of Dye et al. summarizes evidence from basic and clinical research on peripartum changes in both the peripheral and central immune systems, pointing to the need for further research in this area. With regard to the hormones of motherhood, the review of Georgescu et al. highlights the important role of prolactin in shaping the maternal brain and the need to further investigate the role of this key peripartum hormone in developing healthy mother-offspring interactions.

Original research articles of this Research Topic highlight the persistent effects that parenthood has on the parental brain and mental illness. It is well known that sleep can play a role in mental health and brain function but little is known about the link between objective measures of sleep during pregnancy and depressive symptoms. The research of Pitsillos et al. highlights the need for identifying and treating sleep troubles during pregnancy as a preventative measure against perinatal depression. Often environmental factors play a significant role in rates of peripartum mental illness; however, within the United States, a study by Gifford et al. shows that symptom severity in postpartum depression does not differ across 39 states studied, and common social and education factors may play a role in supporting wellbeing of mother and child. Rincón-Cortés and Grace go further and show in a rodent model that social support can partially ameliorate the effects of offspring loss on ventral tegmental area dopamine neuron function, pointing to the need to further understand this brain area in motherhood and pregnancy loss.

Two original articles explore the role of motherhood on fear and anxiety-like behaviors in animal models. Pestana et al. show that pregnancy and not maternal experience has a persistent effect on fear extinction after weaning, and Ragan et al. show that motherhood, but not experience with offspring or levels of anxiety, affect the synthesis of GABA in the medial prefrontal cortex. Thus, it is crucial to consider changes in the maternal brain that are affected by pregnancy itself, in addition to motherhood.

Another original data paper explores the enduring impact of motherhood and mental illness on brain connectivity, this time with depression rather than anxiety. Morgan et al. show that at 12 months postpartum depressive symptoms affect connectivity of the maternal brain and likely play a role in a mother's ability to engage in emotional bonding with her infant.

Finally, an opinion piece titled “Allopregnanolone in Postpartum Depression” highlights the important role that this neurosteroid plays in postpartum depression and calls for further investigation into its neural mechanisms and those of other neurosteroids, suggesting that more research on the neural mechanisms of peripartum mental illness in general is needed. The recent approval of a synthetic form of allopregnanolone for the treatment of postpartum depression highlights the importance of this research area.

Our understanding of the neurobiology of peripartum mental illness is still in its infancy but key findings from current research, highlighted in this Research Topic, point to a number of neural mechanisms that show promise in preventing and treating these debilitating illness—optimizing health and wellbeing for parents, their children and future generations.

## Author Contributions

All authors listed have made a substantial, direct, and intellectual contribution to the work and approved it for publication.

## Conflict of Interest

The authors declare that the research was conducted in the absence of any commercial or financial relationships that could be construed as a potential conflict of interest.

## Publisher's Note

All claims expressed in this article are solely those of the authors and do not necessarily represent those of their affiliated organizations, or those of the publisher, the editors and the reviewers. Any product that may be evaluated in this article, or claim that may be made by its manufacturer, is not guaranteed or endorsed by the publisher.
